# Evaluation of Cellular Immune Responses After mRNA-1273 Vaccination in Children 6 Months to 11 Years of Age

**DOI:** 10.1093/infdis/jiaf144

**Published:** 2025-03-22

**Authors:** Christina A Rostad, James D Campbell, Grant C Paulsen, Sabine Schnyder Ghamloush, Wenqin Xu, Lingyi Zheng, M Juliana McElrath, Stephen C De Rosa, Bethany Girard, Rituparna Das, Evan J Anderson, C Buddy Creech

**Affiliations:** Department of Pediatrics, Emory University School of Medicine and Children's Healthcare of Atlanta, Atlanta, Georgia, USA; Center for Vaccine Development and Global Health, University of Maryland School of Medicine, Baltimore, Maryland, USA; Department of Pediatrics, Division of Pediatric Infectious Diseases, Cincinnati Children’s Hospital Medical Center, University of Cincinnati College of Medicine, Cincinnati, Ohio, USA; Moderna, Inc, Cambridge, Massachusetts, USA; Moderna, Inc, Cambridge, Massachusetts, USA; Moderna, Inc, Cambridge, Massachusetts, USA; Vaccine and Infectious Disease Division, Fred Hutchinson Cancer Center, Seattle, Washington, USA; Vaccine and Infectious Disease Division, Fred Hutchinson Cancer Center, Seattle, Washington, USA; Moderna, Inc, Cambridge, Massachusetts, USA; Moderna, Inc, Cambridge, Massachusetts, USA; Department of Pediatrics, Emory University School of Medicine and Children's Healthcare of Atlanta, Atlanta, Georgia, USA; Moderna, Inc, Cambridge, Massachusetts, USA; Vanderbilt Vaccine Research Program, Vanderbilt University Medical Center, Nashville, Tennessee, USA

**Keywords:** pediatric, COVID-19, SARS-CoV-2, T cells, vaccine

## Abstract

**Background:**

Cell-mediated immunity (CMI) may help protect against emerging severe acute respiratory syndrome coronavirus 2 (SARS-CoV-2) variants that are less susceptible to neutralizing antibodies. We present CMI data after the mRNA-1273 primary series in a subset of participants aged 6 months to 11 years from the phase 2/3 KidCOVE trial.

**Methods:**

T-cell responses were assessed after 2 doses of mRNA-1273 (6 months to 5 years, 25 μg; 6–11 years, 50 μg) or placebo administered 28 days apart. Magnitude, phenotype, and percentage of ancestral SARS-CoV-2 spike (S) protein T-cell responses to pooled peptides were assessed by intracellular cytokine staining and polyfunctionality analyses.

**Results:**

A total of 68 children aged 6 months to 11 years received either the 2-dose mRNA-1273 primary series or placebo (51 and 17, respectively) at 28-day interval. mRNA-1273 induced S-protein–specific CD4^+^ T-cell responses exhibiting a type 1 T helper (Th1)–biased profile at day 43 and day 209 compared with placebo. S-protein–specific CD8^+^ T-cell responses were less frequently detected in children <5 years and undetectable in those <2 years. Compared with placebo, mRNA-1273 induced higher frequencies of S-specific polyfunctional CD4^+^ T cells at day 43; frequencies declined but remained detectable at day 209. Correlation between Th1 CD4^+^ responses and neutralizing antibodies was observed across age groups following mRNA-1273 vaccination.

**Conclusions:**

The 2-dose mRNA-1273 primary series elicited robust and durable (≥ 6 months) Th1-biased CD4^+^ T-cell responses in children aged 6 months to 11 years. CD8^+^ T-cell responses varied by age.

**Clinical Trials Registration.** NCT04796896.

## Teaser

In the phase 2/3 KidCOVE trial, cell-mediated immunogenicity to mRNA-1273 was evaluated in children aged 6 months to 11 years. The 2-dose mRNA-1273 primary series elicited robust and durable Th1-biased CD4^+^ T-cell responses. CD8^+^ T-cell responses varied by age.

Coronavirus disease 2019 (COVID-19) can cause severe respiratory disease among children and adolescents [1]. Vaccination is effective in reducing the risk of SARS-CoV-2 infection [2–5], severe COVID-19 [3, 5, 6], and sequelae, including multisystem inflammatory syndrome in children [7].

COVID-19 vaccination elicits neutralizing antibody (nAb) responses against SARS-CoV-2 [8–10]; however, cell-mediated immune responses also play an integral role and may be capable of protecting against emerging severe acute respiratory syndrome coronavirus 2 (SARS-CoV-2) variants that are less susceptible to nAbs [11–14]. Robust and early T-cell responses following SARS-CoV-2 infection are associated with milder and shorter disease courses, respectively [13], supporting the view that T-cell responses may underlie the protection of COVID-19 vaccines against severe disease [11]. Therefore, induction of robust T-cell responses may be crucial for effective and durable protection against COVID-19, and the magnitude and duration of T-cell responses may help inform the timing of boosters [15–19]. Data on cell-mediated immune responses against SARS-CoV-2 in pediatric populations remain limited. Available data suggest that T-cell responses after infection are lower and decline faster in children compared with adults, with observed age-dependent increases in the magnitude of T-cell responses in children [20, 21].

mRNA-1273 (Spikevax; Moderna, Inc) was developed in response to the COVID-19 pandemic, and was authorized in children based on data from the phase 2/3 KidCOVE trial (Clinical Trials Registration NCT04796896). This trial showed that a 2-dose primary series (25 µg for 6 months to 5 years and 50 µg for 6–11 years age groups) had an acceptable safety profile and induced humoral immune responses consistent with those observed in adolescents and young adults (100-µg doses) [2, 4]. Vaccination with mRNA-1273 was effective against COVID-19 beginning 14 days after the first dose during the delta (vaccine efficacy [VE], 88%) and omicron (VE, 36.8%–50.6%) variant predominance [2, 4].

Analyses of cell-mediated immune responses of mRNA-1273 in adults showed robust and durable memory T-cell responses, and memory B-cell responses postvaccination [22–25]. However, cell-mediated immune responses to mRNA-1273 in pediatric populations remain poorly understood. Here, we report cell-mediated immune responses to mRNA-1273 (primary series) through 6 months after vaccination in a subset of children aged 6 months to 11 years from the KidCOVE trial.

## METHODS

### Trial Design and Participants

The design of this phase 2/3 trial (Clinical Trials Registration NCT04796896) was described previously [2, 4]. Participants (aged 6 months to 11 years) were enrolled at 80 sites in the United States and 8 sites in Canada. Eligible children were generally healthy; children with stable chronic conditions were also included. Additional details on eligibility and trial design have been published previously [2, 4]. The KidCOVE trial includes 3 parts: parts 1 and 2 consisted of an open-label dose-finding phase and an observer-blinded, randomized, placebo-controlled expansion phase, respectively [2, 4]; part 3 was an open-label alternative dosing assessment.

The current analysis of cell-mediated immunity was conducted in a subset of part 2 KidCOVE participants who opted to participate in exploratory analyses. Participants received 2 doses of mRNA-1273 (25 µg for 6 months to 5 years, 50 µg for 6–11 years age groups) or placebo, administered at a 28-day interval, at select Infectious Diseases Clinical Research Consortium sites. Participants were assigned to age groups based on age at enrollment. Data were collected between 9 August 2021 and 7 September 2022 (data cutoff).

### Ethics Statement

The protocol and other relevant documents were approved by the central institutional review board (Advarra, Inc). The study was conducted in accordance with the protocol, applicable laws, and regulatory requirements, the International Council for Harmonization, Good Clinical Practice guidelines, and the ethical principles derived from the Declaration of Helsinki and Council for International Organizations of Medical Sciences International Ethical Guidelines. Participants’ parent(s)/legally authorized representative(s) provided written informed consent and the participant, as applicable, signed the assent form before conduct of study procedures.

### Trial Vaccine

mRNA-1273 is an mRNA-lipid nanoparticle vaccine encoding for the full-length, 2-proline prefusion-stabilized SARS-CoV-2 spike (S) protein [26]. mRNA-1273 was provided as a sterile liquid for injection at 0.2 mg/mL, diluted with normal saline to the appropriate dose, and administered intramuscularly at a dose volume of 0.5 mL at a 28-day interval [2].

### Study Objectives and End Points

The objective of this exploratory analysis was to assess SARS-CoV-2 S-protein–specific T-cell–mediated immune responses. The exploratory end points were the phenotype and percentage of cytokine-expressing S-protein–specific T cells (response magnitude), as well as the proportion of participants with S-protein–specific T-cell responses (response rate), as measured by flow cytometry at different time points after vaccination relative to baseline. Data on the primary safety and immunogenicity end points in parts 1 and 2 have been published [2, 4]. Baseline participant SARS-CoV-2 status was determined as described in the Supplementary Methods. Participants were included in the analysis regardless of baseline SARS-CoV-2 status. All statistical analyses conducted during this study are described in the Supplementary Methods.

### Peripheral Blood Mononuclear Cell Sample Processing

Peripheral blood mononuclear cells (PBMCs) were collected from participants at baseline (day 1), 14 days (day 43), and 180 days (day 209) after dose 2 (which was administered on day 29), via a preapproved protocol across all sites participating in PBMC processing. As the need for a 2-dose primary mRNA-1273 vaccinations series has previously been established in phase 1 trials in adults [22, 26], days 43 and 209 were selected to focus on the time period anticipated to display T-cell responses and durability. One to 1.2 million PBMCs were stimulated for each condition; if insufficient cells were recovered, then some stimulation conditions were dropped rather than decreasing the number of cells to maintain sensitivity for detection of low-level responses.

### Intracellular Cytokine Stimulation Assay

The SARS-CoV-2 S-protein–specific CD4^+^ and CD8^+^ T-cell response rates as well as the phenotypes and percentage of responses were measured using a validated intracellular cytokine staining assay as described previously [27, 28]. Peptide pools covering the S protein of SARS-CoV-2 (316 peptides covering the original Wuhan strain with 4 peptides covering the D614G variant) were synthesized as 15 amino acids overlapping by 11 amino acids (BioSynthesis). The peptides were distributed into an S1 pool (173 peptides) and an S2 pool (147 peptides); the final concentration of each peptide was 1 µg/mL. PBMC were stimulated for 6 hours with either the peptide pools or a polyclonal stimulant (staphylococcal enterotoxin B) as a positive control. Antigen-specific cytokine frequencies were reported after background subtraction of identical gates from the same sample incubated with the negative control stimulation (0.5% dimethyl sulfoxide peptide diluent) [27]. Samples were analyzed using a BD FACSymphony A5 flow cytometer (BD Biosciences) with a 5-laser configuration. Data were analyzed in a blinded fashion using standardized templates in FlowJo version 9.9.4 (BD Biosciences). Gating details and threshold limits for T-cell activation are provided in the Supplementary Methods.

### Polyfunctional Analysis

The combinatorial polyfunctionality analysis of antigen-specific T-cell subsets (COMPASS) uses a Bayesian hierarchical mixture model to identify antigen-specific changes in all T-cell subsets simultaneously [29]. This model was used to analyze CD4^+^ and CD8^+^ T-cell responses to the S1 and S2 peptide pool, although for the present analysis, the magnitudes of responses rather than probabilities of antigen-specific responses are shown in heatmaps. The heatmap represents different subsets of cytokines in columns, grouped from left to right based on their increasing number of functions [30].

### Neutralizing Antibody Analysis

Residual plasma from PBMC processing was tested in a pseudovirus neutralization assay (PsVNA) developed at PPD Laboratories against the SARS-CoV-2 S protein [31]. In brief, 293T-ACE2 cells were seeded according to vendor standard operating procedures, then serum samples were thawed and incubated for 1 hour with SARS-CoV-2 S (D614G) reporter virus particles, which express green fluorescent protein (GFP). The seeded 293T-ACE2 cells were infected with the preincubated plasma and SARS-CoV-2–GFP for 48 hours. Serum samples were tested in duplicate, with each replicate on a separate plate in a dilution series of 1:10 or 1:50, or a 1:16 predilution series. After 48 hours of culture, the cell plates were fixed and GFP foci were detected with an assay read buffer.

### Neutralizing Antibody Correlation Analysis

nAb titers against SARS-CoV-2 protein (strain D614G) in the mRNA-1273 or placebo groups, as measured by the PsVNA [31], were plotted against the frequencies of S-protein–specific Th1 CD4^+^ T cells, as measured by the intracellular cytokine staining assay. The correlation between these 2 assays was assessed by Pearson correlation coefficient of log-transformed results and Spearman rank correlation coefficient.

## RESULTS

### Participants

**Figure 1.**
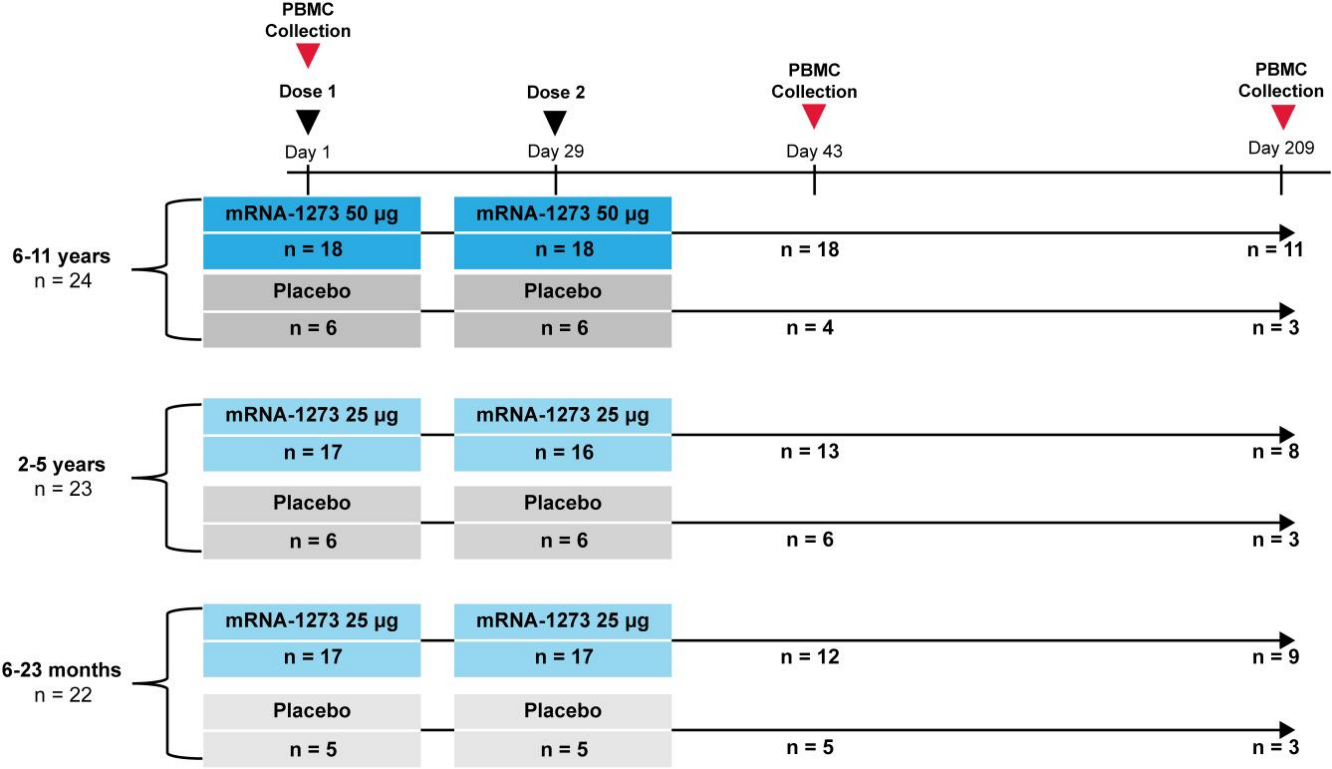
Study design. The flow of participants by vaccine group and schedule of blood collection for analysis in the exploratory cohort. Eligible participants received a 2-dose primary series of mRNA-1273 (aged 6 months to 5 years, 25 µg; 6–11 years, 50 µg) or placebo. Blood collections for the exploratory analysis occurred at day 1 (baseline, prevaccination), day 43 (14 days after dose 2), and day 209 (180 days after dose 2). Data cutoff was 7 September 2022. Abbreviation: PBMC, peripheral blood mononuclear cell.

The overall disposition and demographics of KidCOVE participants was described previously [2, 4]. In this exploratory analysis, a total of 69 part 2 participants aged 6 to 23 months (n = 22); 2 to 5 years (n = 23); and 6 to 11 years (n = 24) were randomly assigned to receive a 2-dose primary series regimen of mRNA-1273 (25 µg for 6 months to 5 years; 50 µg for 6–11 years) or placebo in a 3:1 ratio (Figure 1). Among those randomized, 68 participants (6–23 months [n = 22]; 2–5 years [n = 22]; and 6–11 years [n = 24]) received both doses of mRNA-1273 or placebo (51 and 17, respectively) and were included in the analysis (Figure 1). The median age by group was 15 months (interquartile range [IQR], 10–19 months), 2 years (IQR, 2–3 years), and 8 years (IQR, 7–10 years). At baseline, all participants aged 6–23 months, and 6–11 years were negative for recent or prior SARS-CoV-2 infection (reverse transcription polymerase chain reaction [RT-PCR] and serology). Among participants aged 2–5 years at baseline, 6 had no results available, 3 had positive RT-PCR and serology results, and 14 were seronegative. During the study period, 28 participants experienced RT-PCR−confirmed intercurrent infection.

### SARS-CoV-2 S-Specific T-Cell Responses to mRNA-1273 Vaccination

**Figure 2.**
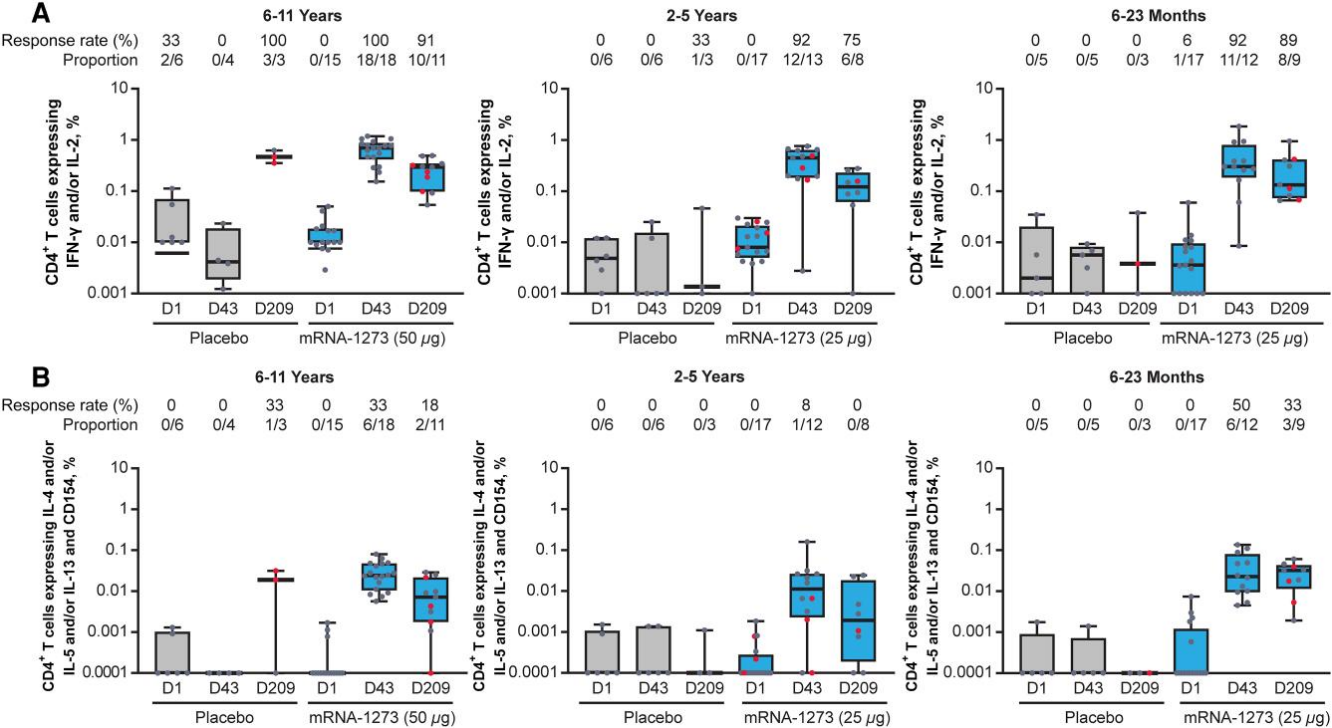
S-specific CD4^+^ T cells exhibiting (*A*) Th1 and (*B*) Th2 profiles among children aged 6 months to 11 years. Frequencies of Th1 CD4^+^ T cells (expressing IFN-γ and/or IL-2) or Th2 CD4^+^ T cells (expressing IL-4 and/or IL-5 and/or IL-13 and CD154) in mRNA-1273 or placebo recipient groups following ex vivo stimulation with SARS-CoV-2 S1 + S2 peptide pools as measured by flow cytometry. Time points include D1 (baseline/dose 1), D43 (14 days after dose 2), and D209 (180 days after dose 2) for the 6–11 years (left), 2–5 years (middle), and 6 to 23 months (right) age groups. Response rates (proportion of participants with a positive S-specific CD4^+^ T-cell response) are shown at the top of each graph. Cytokine-expressing cells were determined by gating on singlets, lymphocytes, viability dye-CD3^+^, followed by CD4^+^ or CD8^+^ (additional details in the Supplementary Methods). Horizontal lines within boxes indicate median values, vertical bars span the lower quartile to upper quartile, and whiskers span the minimum to maximum. Red dots indicate nucleocapsid seropositive individuals: 6–11 years (placebo, D209 = 2; mRNA-1273, D209 = 4), 2–5 years (mRNA-1273, D1 = 3, D43 = 3, D209 = 1), and 6–23 months (placebo, D209 = 1; mRNA-1273: D209 = 3). Seropositivity defined as participants who tested positive using the Elecsys Anti-SARS-CoV-2 assay (Roche), which uses recombinant nucleocapsid antigen for the determination of SARS-CoV-2-specific antibodies. Abbreviations: D, day; IFN, interferon; IL, interleukin; S, spike; Th, T helper cell.

S-specific CD4^+^ and CD8^+^ T-cell responses to the 2-dose mRNA-1273 (primary series) were assessed using a validated intracellular cytokine staining assay following 6-hour stimulation with the S1 and S2 peptide pools. Prior to vaccination, type 1 T helper (Th1)-biased CD4^+^ T-cell responses (expressing interferon-γ [IFN-γ] and/or interleukin 2 [IL-2]) were detected in 1 participant in the mRNA-1273 group (6–23 months) and 2 participants in the placebo group (6–11 years); all 3 participants were negative for active and prior SARS-CoV-2 infection at baseline (RT-PCR and serology). mRNA-1273 vaccination induced robust Th1 CD4^+^ T-cell responses, with 92% to 100% of mRNA-1273 recipients across age groups exhibiting responses at 14 days following dose 2 (day 43) versus 0% after placebo (Figure 2*A*). Th1 CD4^+^ responses after mRNA-1273 vaccination declined at day 209 but remained substantially above baseline in all age groups, with 75% to 91% of participants exhibiting durable responses. Similar results were seen in response to tumor necrosis factor (TNF) expression (Supplementary Figure 1). In contrast, type 2 T helper (Th2) CD4^+^ T-cell responses (expressing IL-4, IL-5, and/or IL-13) at day 43 were present in 50% (6–23 months), 8% (2–5 years), and 33% (6–11 years) of mRNA-1273 recipients versus 0% after placebo (Figure 2*B*). Th2 CD4^+^ response magnitudes were below those observed for Th1, and generally declined in all age groups, with 33% (6–23 months), 0% (2–5 years), and 18% (6–11 years) of mRNA-1273 recipients with responses at day 209 (Figure 2*B*). The median CD4^+^ Th2 response was markedly lower compared with the median CD4^+^ Th1 response on day 43 across all age groups (6–23 months, 0.023 vs 0.30; 2–5 years, 0.011 vs 0.45; 6–11 years, 0.024 vs 0.69; Figure 2). The CD4^+^ T-cell responses remained Th1-biased in all age groups through day 209 (Supplementary Figure 2).

**Figure 3.**
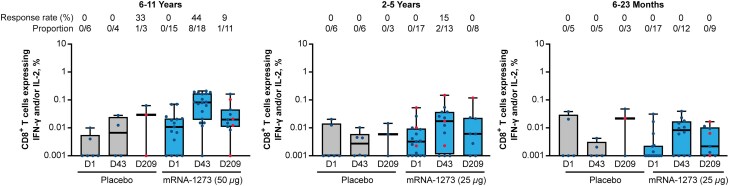
S-specific CD8^+^ T-cell responses among children aged 6 months to 11 years. Frequencies of CD8^+^ T cells (expressing IFN-γ and/or IL-2) in mRNA-1273 or placebo groups following ex vivo stimulation with SARS-CoV-2 S1 + S2 peptide pools as measured by flow cytometry. Time points include D1 (baseline/dose 1), D43 (14 days after dose 2), and D209 (180 days after dose 2) for the 6–11 years (left), 2–5 years (middle), and 6–23 months (right) age groups. Response rates (proportion of participants with positive CD8^+^ T-cell response) are shown at the top of each graph. Cytokine-expressing cells were determined by gating on singlets, lymphocytes, viability dye-CD3^+^, followed by CD4^+^ or CD8^+^ (additional details in the Supplementary Methods). Horizontal lines within boxes indicate median values, vertical bars span the lower quartile to upper quartile, and whiskers span the minimum to maximum. Red dots indicate nucleocapsid seropositive individuals for 6–11 years (placebo, D209 = 2; mRNA-1273, D209 = 4), 2–5 years (mRNA-1273, D1 = 3, D43 = 3, D209 = 1), and 6–23 months (placebo, D209 = 1; mRNA-1273, D209 = 3). Seropositivity defined as participants who tested positive using the Elecsys Anti-SARS-CoV-2 assay (Roche), which uses recombinant nucleocapsid antigen for the determination of SARS-CoV-2-specific antibodies. Abbreviations: D, day; IFN, interferon; IL, interleukin; S, spike.

S-specific CD8^+^ T-cell responses (expressing IFN-γ and/or IL-2) to mRNA-1273 vaccination were lower in magnitude than CD4^+^ Th1 responses and were dependent on participant age (Figure 3). Prior to vaccination with placebo or mRNA-1273, CD8^+^ T-cell responses were not observed. In the youngest age group (6–23 months), no antigen-specific CD8^+^ T cells were detected at day 43 or day 209 for either mRNA-1273 or placebo recipients. Among those aged 2 to 5 years, 15% of mRNA-1273 recipients had CD8^+^ T-cell responses at day 43, which declined to baseline (0%) by day 209; no responses were detected in placebo recipients. CD8^+^ T-cell responses were detected in 44% of older children (6–11 years) at day 43 and in 9% at day 209; notably, 1 placebo recipient (1 of 3 participants; 33%) aged 6 to 11 years had T-cell responses at day 209 only (Figure 3).

### Polyfunctional T-Cell Responses to mRNA-1273 Vaccination

**Figure 4.**
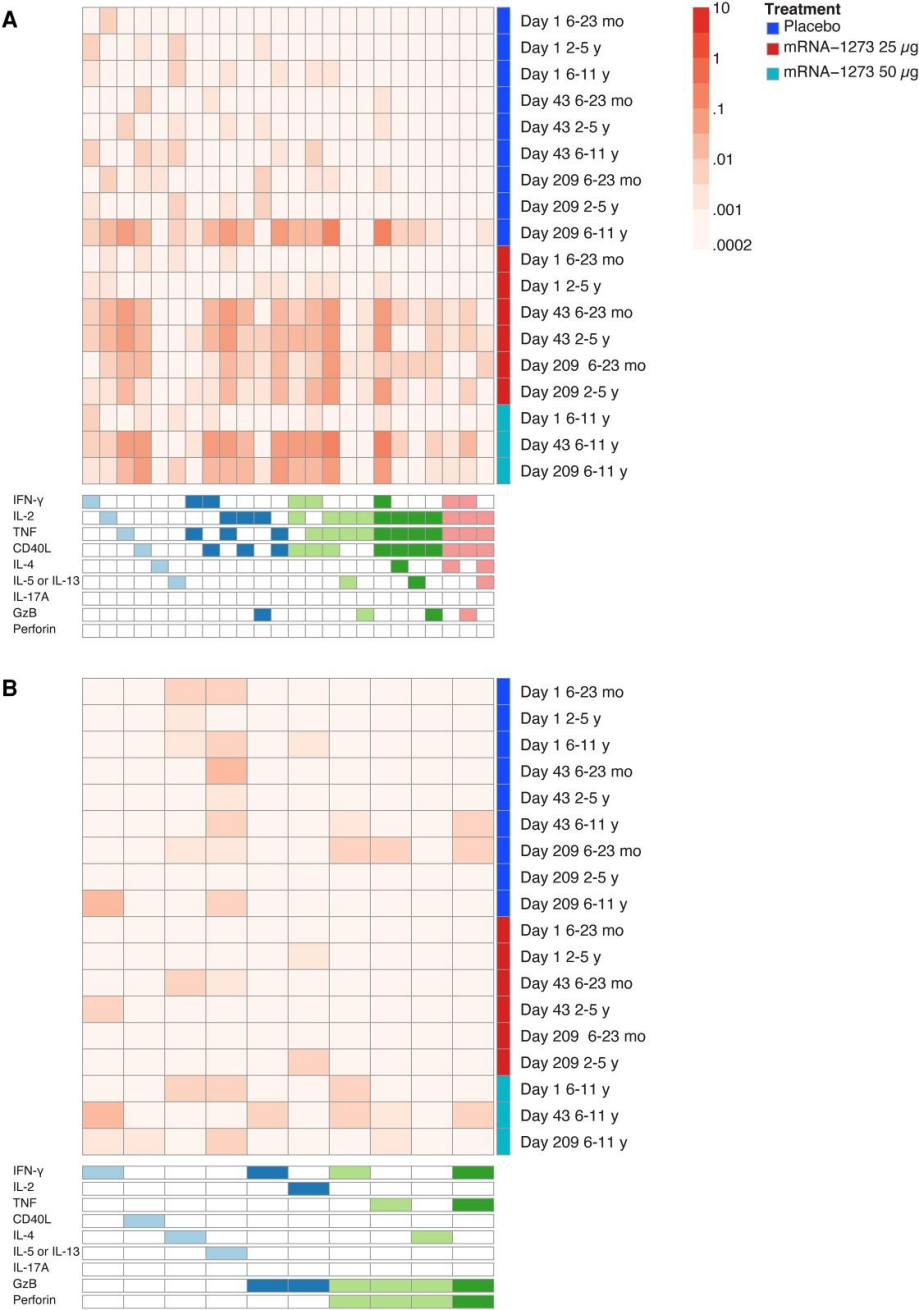
Polyfunctional analysis of (*A*) CD4^+^ or (*B*) CD8^+^ T-cell responses in participants using S1 and S2 peptide pools. Heatmap for CD4^+^ or CD8^+^ T-cell responses to the S1 and S2 peptide pools showing the median background-adjusted magnitude of antigen-specific responses from each of the cell subsets expressing various combinations of functional markers determined by COMPASS. A filled box in each column indicates cells expressing that function, with different colors depicting the number of functions (1 to 5) expressed. Columns correspond to the different cell subsets, and the key below the heatmap is color-coded and ordered by degree of functionality from 1 function on the left (light blue boxes) to 5 functions on the right (pink boxes). Rows correspond to the treatment group (placebo [blue boxes], mRNA-1273 25 µg [red boxes], and mRNA-1273 50 µg [teal boxes]) and age group at each time point. Each cell in the heatmap shows the background-adjusted median magnitude in the respective treatment group (row) for the corresponding antigen-specific subset (column) where the magnitude is color-coded from 0.0002% to 10%. Abbreviations: COMPASS, Combinatorial Polyfunctionality Analysis of Antigen-Specific T-Cell Subsets; GzB, granzyme B; IFN, interferon; IL, interleukin; TNF, tumor necrosis factor.

Polyfunctionality analysis of CD4^+^ and CD8^+^ T-cell responses to the S1 and S2 peptide pools was performed using COMPASS. Compared with placebo, mRNA-1273 vaccination induced higher frequencies of S-specific polyfunctional CD4^+^ T cells (coexpressing IFN-γ, IL-2, TNF, and CD40L) at day 43 for all age groups (Figure 4*A*). Frequencies of these polyfunctional CD4^+^ T cells declined but remained detectable at day 209 for all vaccine groups. Among participants who received placebo, polyfunctional responses were largely only detected at day 209 in those aged 6 to 11 years. This was likely due to SARS-CoV-2 infection during the study; at day 209, 2 of 3 participants were confirmed as seropositive and 1 participant was seronegative. Cells expressing 4 functional markers (IL-4 or IL-5, with IL-2 and TNF, but not IFN-γ) were detected at lower frequencies. Low frequencies of 4- and 5-function cells including granzyme B were also detected. Of the 9 evaluated CD4^+^ functional markers, only IL-17A and perforin were not detected among the responding cells of any vaccine group. S-specific CD8^+^ T cells were generally less polyfunctional than the CD4^+^ T cells. Four-function cells expressing IFN-γ, TNF, granzyme B, and perforin, and 3-function cells expressing granzyme B and perforin with either IFN-γ or TNF were detected mainly in the older age group (Figure 4*B*).

### Correlation to Neutralizing Antibody Responses

**Figure 5.**
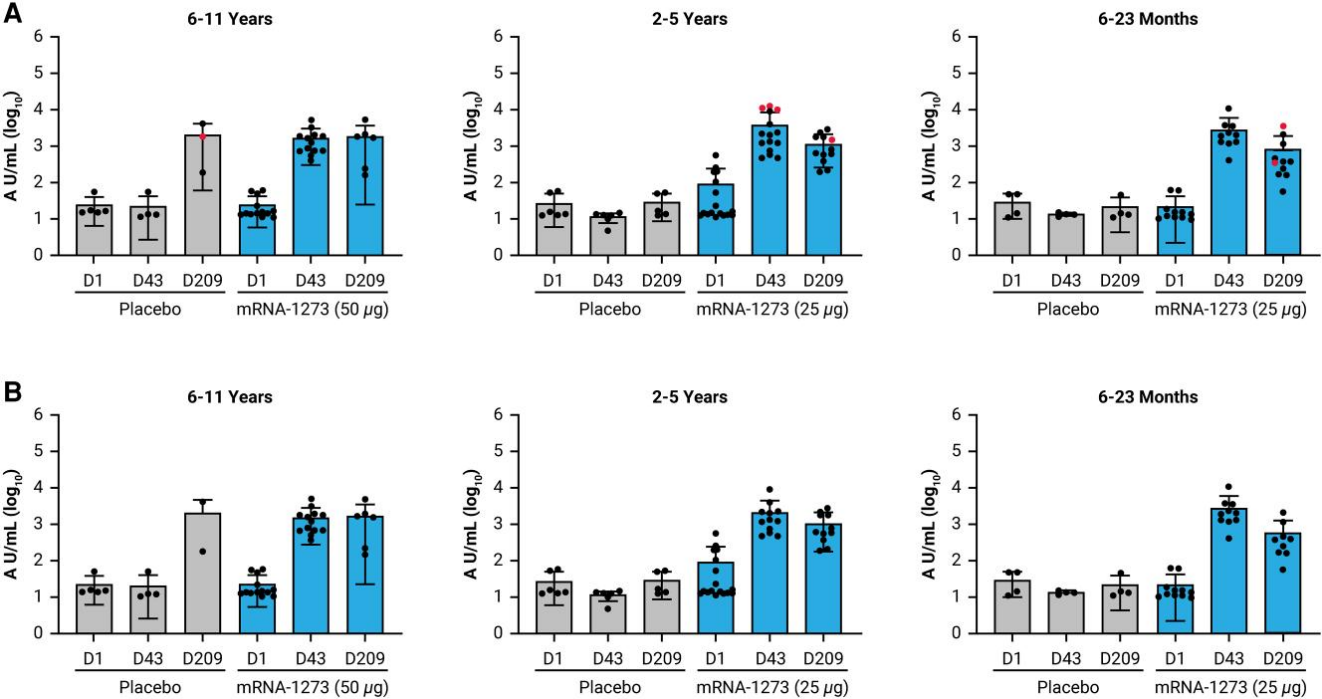
nAb concentration against SARS-CoV-2 among children aged 6 months to 11 years: (*A*) overall, and (*B*) seronegative subset. nAbs against SARS-CoV-2 in mRNA-1273 or placebo groups as measured by PsVNA against SARS-CoV-2 S protein (strain D614G). Time points include D1 (baseline/dose 1), D43 (14 days after dose 2), and D209 (180 days after dose 2) for 6–11 years (left), 2–5 years (middle), and 6–23 months (right) age groups. Bars indicate the geometric means and vertical lines span the lower and upper bounds of the 95% confidence interval. Red dots indicate nucleocapsid seropositive individuals for 6–11 years (placebo, D209 = 1), 2–5 years (mRNA-1273, D1 = 3, D43 = 3, D209 = 1), and 6–23 months (mRNA-1273, D209 = 2). Seropositivity defined as participants who tested positive using the Elecsys Anti-SARS-CoV-2 assay (Roche), which uses recombinant nucleocapsid antigen for the determination of SARS-CoV-2-specific antibodies. In some cases, not all PBMCs had residual plasma available for analysis. Some participants had residual plasma available for analysis, but PBMCs were not successfully analyzed due to low cell count/viability. Abbreviations: AU, arbitrary units; D, day; nAb, neutralizing antibody; PBMC, peripheral blood mononuclear cell; PsVNA, pseudovirus neutralization assay; SARS-CoV-2, severe acute respiratory syndrome coronavirus 2.

Across all age groups, increased nAb responses against SARS-CoV-2 S protein (D614G) at day 43 and day 209 were observed in participants in the mRNA-1273 group compared with those in the placebo group where nAb responses remained similar to or decreased from baseline at corresponding time points (Figure 5). One exception was the result at day 209 in participants aged 6 to 11 years, which may have been attributable to the Omicron surge observed in the study between the day 43 and day 209 time points. The increased nAb responses in mRNA-1273 recipients correlated with the CD4^+^ T-cell response across all age cohorts (moderate to high correlation), while there was minimal correlation with the placebo group (Supplementary Figure 3). The overall Pearson correlation coefficient and Spearman rank correlation coefficient were 0.7480 and 0.7326, respectively, for all vaccine recipients combined. Although intercurrent SARS-CoV-2 infection was observed among 28 participants during the study, nAb responses were similar among all participants and among seronegative participants (Figure 5). Collectively, these results demonstrate that mRNA-1273 generates cellular and humoral responses in children.

## DISCUSSION

Previous findings from the phase 2/3 KidCOVE trial demonstrated that a 2-dose primary series of mRNA-1273 in children (25 µg for 6 months to 5 years and 50 µg for 6–11 years age groups) had an acceptable safety profile and elicited humoral immune responses consistent with those observed in adolescents and young adults (100-µg dose) [2, 4]. Here, we report cell-mediated immune responses through 6 months after mRNA-1273 primary series vaccination in a cohort of KidCOVE participants.

The 2-dose primary series induced robust S-specific CD4^+^ T-cell responses in participants 6 months to 11 years of age, primarily exhibiting a Th1-biased profile 14 days after dose 2 that was sustained ≥ 6 months. These CD4^+^ T-cell responses were highly polyfunctional, with cells coexpressing combinations of multiple functional markers. CD8^+^ T-cell responses at day 43 were less robust than CD4^+^ and were age-dependent, with responses detected principally in older age groups. These findings are consistent with previous observations of strongly biased CD4^+^ Th1 response and lower CD8^+^ responses following vaccination with mRNA-1273 in both healthy younger [23, 26] and older adults [22]. nAb responses correlated with CD4^+^ responses in all vaccine groups. As expected, nAb responses remained low for participants in the placebo group, except for the 6–11 years group at day 209.

As cell-mediated responses likely play an important role in protecting against severe outcomes from SARS-CoV-2 in adults, it is plausible that the CD4^+^ responses demonstrated here would confer similar protection in children [32]. The importance of these findings is underpinned by data showing that cell-mediated immunity induced by mRNA-1273 in adults is less affected by SARS-CoV-2 variants, including Omicron [33], which can evade nAb responses. nAbs bind to epitopes on the SARS-CoV-2 spike protein and block its engagement with the host cell, leading to immune pressure for these epitopes to evolve and evade this protective mechanism [34]. In contrast, T cells bind to 8- to 15-amino acid linear peptides presented on the major histocompatibility complex I (MHC-I) or MHC-II of host cells, limiting viral propagation within the host and blunting disease severity. This property renders T-cell immunity less susceptible to immune evasion attributable to mutation of neutralizing epitopes and may explain its resilience against emerging variants [34].

Highly functional memory T cells are characterized by an ability to express multiple cytokines simultaneously, suggesting a broad and robust immune response [35]. Cytokines that drive the maturation of T helper cells influence critical transcription factors that specify various cell lineages and induce T-cell differentiation and function as negative regulators for other cell subsets [36]. Therefore, the cytokine environment is essential for functional diversity and adaptability of memory T cells. Importantly, Th1 and Th2 CD4^+^ T cells can develop into long-lived central and effector memory cells that retain functions consistent with their initial differentiation, even though some flexibility may be present in their cytokine expression profiles [37, 38]. As the Omicron variant continues to evolve immunoevasive sublineages, including recent variants of interest JN.1 and KP.3, understanding the protective properties of vaccine-induced T-cell responses remains imperative for pediatric populations [39, 40]. Updated variant-targeting mRNA-1273 vaccines are now authorized for use in multiple countries; these have been developed to broaden protection by inducing variant-specific nAb responses [41–44]. The results presented here build upon the knowledge of mRNA-1273 vaccination in children [4] to include younger participants (6 months to <6 years) and shed light on cell-mediated responses in pediatric populations. Additionally, the characterization of antigen-specific T-cell cytokine profiles using COMPASS expands on findings from previous studies by providing a more granular view of T-cell functionality after mRNA vaccination in children [45–47]. Together, these results further support the use of the mRNA-based platform in pediatric populations to protect against COVID-19 [2].

This report raises important considerations when assessing vaccine-induced cell-mediated immune responses, especially considering antigenic complexity of the SARS-CoV-2 virus coupled with the evolving immunity of pediatric age groups evaluated here [48]. Notably, CD4^+^ (Th1 and Th2) and CD8^+^ responses were observed at day 209 among some placebo recipients (6–11 years), which may be attributable to intercurrent SARS-CoV-2 infection between days 43 and 209. Of note, low-level T-cell responses to the S2 pool may represent previous or intercurrent exposure to other human beta-coronaviruses [49]. In contrast to CD4^+^ responses, the CD8^+^ responses to mRNA-1273 vaccination appeared age-dependent, with no observable response in children 6–23 months and with increasing responses in children aged 2–5 years and 6–11 years. This aligns with previous reports of T-cell responses to SARS-CoV-2 infection being lower in children than in adults [50]. Interestingly, it has previously been shown that percentages of CD8^+^ T-cell levels follow a similar pattern in healthy individuals, starting low in infants (1–7 months) and increasing gradually through adulthood (21–50 years), before declining again in older adults (70–92 years) [48]. Further, CD4^+^ T-cell levels start much higher in infants when compared with CD8^+^ T-cell levels, followed by a decrease and plateau in children aged 1 to 18 years [48]. Additional data are needed to understand the relevance of low S-specific CD8^+^ T-cell responses in younger children across various vaccine platforms.

This study is among the first clinical trial evaluations of cell-mediated immunity in children, which is important to gain understanding of the immune response to vaccination compared with natural infection. Additionally, the use of standardized, validated assays for evaluating T-cell responses to SARS-CoV-2 strengthens the present data. Limitations of this study include the exploratory nature of the analysis focusing on a subset of trial participants, thus prohibiting statistical comparisons and broader conclusions across age groups, especially given the potential variation between immune system activity between infants and older children [48]. The presence of infection in a subset of participants and missing data at certain time points further complicates the interpretation of results. Additionally, this study evaluated the ancestral strain of SARS-CoV-2 and the data are not matched to the currently recommended variant-adapted vaccine. Understanding the relative contribution of cellular immunity to protection from COVID-19, especially to emerging SARS-CoV-2 variants, is an important area for future research.

In conclusion, the 2-dose mRNA-1273 primary series elicited robust Th1-biased CD4^+^ T-cell responses that were durable through ≥ 6 months in children 6 months to 11 years of age. Age-dependent CD8^+^ T-cell responses were also observed. These findings highlight the importance cell-mediated immune responses against SARS-CoV-2 and support the further evaluation of mRNA-based COVID-19 vaccination in children.

## Supplementary Material

jiaf144_Supplementary_Data

## Supplementary Data

Supplementary materials are available at *The Journal of Infectious Diseases* online (http://jid.oxfordjournals.org/). Supplementary materials consist of data provided by the author that are published to benefit the reader. The posted materials are not copyedited. The contents of all supplementary data are the sole responsibility of the authors. Questions or messages regarding errors should be addressed to the author.

## Notes

***Author contributions.*** B. G., S. S. G., R. D., E. J. A., S. C. D. R., and C. B. C. conceived and designed the study. C. A. R, J. D. C., G. C. P., B. G., M. J. M., S. C. D. R., E. J. A., R. D., and C. B. C. collected the data. C. A. R., G. C. P., L. Z., W. X., B. G., S. S. G., M. J. M., S. C. D. R., E. J. A., R. D., and C. B. C. analyzed and interpreted the data. All authors contributed to the drafting and review of the manuscript, and approved the final version of the manuscript.

***Acknowledgments.*** Medical writing and editorial assistance were provided by Andy Kerr, PhD, Jennifer McKinney, PhD, and Ashlea Inan, PhD, of MEDiSTRAVA in accordance with Good Publication Practice (GPP 2022) guidelines, funded by Moderna, Inc, and under the direction of the authors. We thank Kathy Stephens, MSN and the clinical research team at Emory Children's Center-Vaccine Research Clinic for their contributions to this study, as well as the clinical research staff of the Vanderbilt Vaccine Research Program in Nashville, TN. We thank the pediatric clinical research team at the University of Maryland School of Medicine Center for Vaccine Development and Global Health. We thank the following people in the McElrath laboratory at the Fred Hutchinson Cancer Center: Meron Abay (Lead Research Technician), Katharine Schwedhelm (Laboratory Research Manager), Daryl Morris (Statistician), Greg Mize (QA), Todd Haight (Repository Manager), and Carol Marty (Data Manager). We thank the study participants and families for making this study possible.

***Data availability.*** Access to participant-level data presented in the article and supporting clinical documents by qualified external researchers who provide methodologically sound scientific proposals may be available upon reasonable request for products or indications that have been approved by regulators in the relevant markets and subject to review from 24 months after study completion. Such requests can be made to Moderna, Inc, 325 Binney Street, Cambridge, MA 02142; data_sharing@modernatx.com. A materials transfer and/or data access agreement with the sponsor will be required for accessing shared data. All other relevant data are presented in the article. This protocol is available online at ClinicalTrials.gov: NCT04796896.

***Financial support***. This work was supported by federal funds from the Department of Health and Human Services, Administration for Strategic Preparedness and Response, Biomedical Advanced Research and Development Authority (contract number 75A50120C00034 with Moderna, Inc.); the Infectious Diseases Clinical Research Consortium through the National Institute of Allergy and Infectious Diseases, National Institutes of Health (NIAID, NIH) (grant number UM1AI148684); the HIV Vaccine Trials Network (grant number UM1AI068618); and NIAID, NIH (grant number UM1 AI068618 to M. J. M. and S. C. D. R.). The content is solely the responsibility of the authors and does not necessarily represent the official views of the National Institutes of Health.
