# Supplementary material for: Evaluation of Cellular Immune Responses After mRNA-1273 Vaccination in Children 6 Months to 11 Years of Age
Source: J Infect Dis. 2025 Mar 22;231(5):e945–55. doi: 10.1093/infdis/jiaf144 (PMC12128075; doi:10.1093/infdis/jiaf144)
Supplement: jiaf144_Supplementary_Data [file jiaf144_supplementary_data.docx]

Supplement

# List of KidCOVE CMI Study Group Investigators

| **Affiliation** | **Principal Investigator(s)** | **Study Group** | **Location** |
| --- | --- | --- | --- |
| Emory University School of Medicine | Christina A. Rostad, MD  Evan J. Anderson, MD | Satoshi Kamidani, MD | Atlanta, GA |
| University of Maryland School of Medicine | James D. Campbell, MD, MS | Elizabeth Hammershaimb, MD Ginny Cummings, CRNP Andrea Berry, MD  Karen Kotloff, MD | Baltimore, MD |
| Cincinnati Children’s Hospital Medical Center | Grant C. Paulsen, MD | Robert W. Frenck, Jr., MD | Cincinnati, OH |
| Vanderbilt University Medical Center | C. Buddy Creech, MD, MPH | Stephanie Rolsma, MD, PhD  Shannon Walker, MD | Nashville, TN |

# Supplementary Methods

*Determination of prior or recent SARS-CoV-2 infection*

SARS-CoV-2 infection status at baseline was determined by collection of a nasal swab and analysis by reverse transcriptase polymerase-chain-reaction (RT-PCR) for the qualitative detection of nucleic acid from SARS-CoV-2, with positive results being indicative of the presence of SARS-CoV-2 RNA. Additionally, serology testing was also performed at baseline using the Elecsys nucleocapsid binding immunoassay (Roche), with positive result, indicating recent or prior infection.

## Peripheral blood mononuclear cell sample processing

Whole blood was collected from participants directly into cell preparation tubes containing sodium citrate additive and transported to the laboratory. Tubes were centrifuged at room temperature for 30 minutes at 1800 x g relative centrifugal force, after which the plasma was removed and aliquoted before the mononuclear cell layer was harvested and transferred to a 50-mL conical tube. PBMCs were washed 3 times with Ca^2+^/Mg^2+^-free phosphate buffered solution (PBS) (Fisher Scientific, Cat. #BW17-516Q), counted via automated cell counter, and cryopreserved at 5 million cells/mL per tube, maintained in liquid nitrogen storage until analysis

## Intracellular cytokine stimulation assay

The threshold for T-cell activation was consistent for all groups. Assay data were deemed acceptable if the total CD4+ and CD8+ T-cell numbers exceeded 10,000 and 5000, respectively. Positivity was determined using a one-sided Fisher’s exact test. A multiplicity adjustment was made across all tested peptide pools using the discrete Bonferroni adjustment method. If the adjusted p-value for an antigen was ≤10^-5^, the response to the antigen was considered positive for the T-cell subset. If either or both S1 and S2 peptide pools were positive for a T-cell subset, then the total spike response for that T-cell subset was considered positive. The overall response to SARS-CoV-2 spike was defined as the sum of the background-subtracted responses to each of the individual pools.

Cytokine-positive cells were determined by gating on singlets, lymphocytes, viability dye-CD3+, followed by CD4+ or CD8+. Individual cytokines were plotted on the y-axis versus CD69 on the x-axis, and only the CD69+cytokine+ events were used to determine positive responses. Positive cytokine gates were determined using unstimulated samples during qualification testing. A template of gating was created during assay qualification and was applied to all vaccine samples without manipulation. “Any responses” are any combination of the indicated individual cytokines by a population of CD4+ or CD8+ T cells and were calculated using Boolean combination gates. All antigen-specific cytokine frequencies were reported after background subtraction of identical gates from the same sample incubated with the negative control stimulation (DMSO). Example plots of the flow cytometric gating strategy are shown in **Supplementary Figure 4.**

## Statistical analysis

The positivity for a peptide pool within a T-cell subset was assessed by constructing a 2-by-2 contingency table to compare the peptide stimulated and negative control data for each cytokine subset. The entries in each table represented the numbers of cells that were positive and negative for expression of the cytokine(s) following both the peptide stimulation and negative control. If both negative control replicates were included, the average for the total number of cells and the average for the number of positive cells were used. A one-sided Fisher’s exact test was applied to determine whether the number of cytokine-expressing cells for the peptide stimulated data was equal to that for the negative control. Multiple individual tests were conducted simultaneously for each peptide pool; therefore, a multiplicity adjustment was applied to the p values of individual peptide pools using the Bonferroni-Holm adjustment method. If the adjusted p value for a peptide pool was ≤0.00001, the response to the peptide pool for the T-cell subset was considered positive. Due to the large total cell counts for the T-cell subsets (eg, 100,000 cells), the Fisher’s exact test had high power to reject the null hypothesis for very small differences. Therefore, the adjusted p value significance threshold of ≤0.00001 was chosen [28]. The overall response to S protein was considered positive if ≥1 S protein peptide pool (S1 or S2) was positive for the T-cell subset, and the magnitude is the sum of the frequencies of the S1 and S2 pool responses.

# Supplementary Figures

**
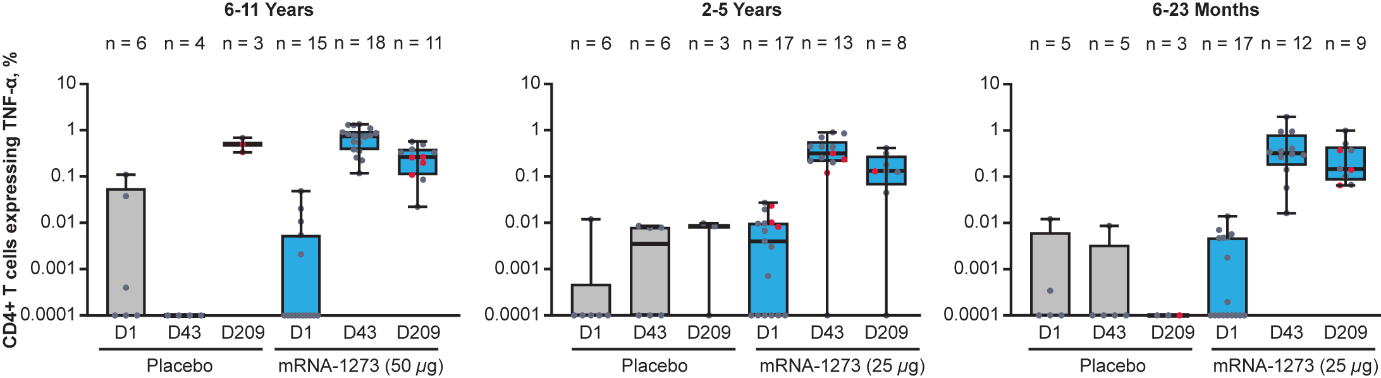
**

**Figure S1. CD4+ T-cell responses to SARS-CoV-2 S protein TNF expression among children aged 6 months to 11 years.** Frequencies of CD4+ T cells (producing IFN-α) in mRNA-1273 or placebo groups following ex vivo stimulation with SARS-CoV-2 S1+S2 peptide pools as measured by flow cytometry. Time points include D1 (Baseline/Dose 1), D43 (14 days PD 2), and D209 (180 days PD 2) for the 6 to 11 years (left panels), 2 to 5 years (middle panels), and 6 to 23 months (right panels) age groups. Numbers of participants with CD4+ T-cell response are shown at the top of each graph. Cytokine-positive cells were determined by gating on singlets, lymphocytes, viability dye-CD3+, followed by CD4+ or CD8+ (additional details in the **Supplementary Methods**). Horizontal lines within boxes indicate median values and vertical bars span the lower quartile (minimum) and upper quartile (maximum). Red dots indicate nucleocapsid seropositive individuals: 6 to 11 years (Placebo: D209 = 2; mRNA-1273: D209 = 4), 2 to 5 years (mRNA-1273: D1 = 3, D43 = 3, D209 = 1), and 6 to 23 months (Placebo: D209 = 1; mRNA-1273: D209 = 3). Seropositivity defined as participants who tested positive using the Elecsys Anti-SARS-CoV-2 assay (Roche), which uses recombinant nucleocapsid antigen for the determination of SARS-CoV-2‒specific antibodies.
*Abbreviations*: D, Day; PD, post-dose; S, spike; TNF; tumor necrosis factor.

**
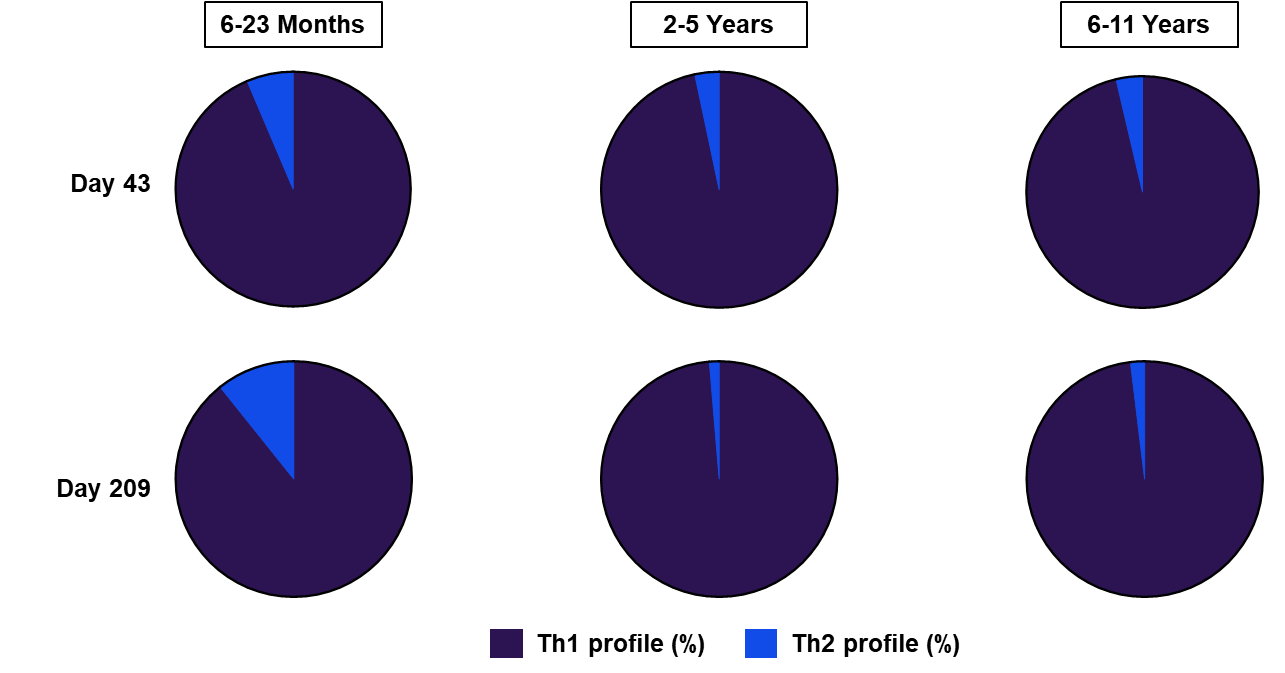
**

**Figure S2. Th1:Th2 balance as a proportion of the total S protein-specific T-cell response.** Pie charts represent the % median CD4+ T cells that exhibited a Th1 profile (IFN- ɣ and/or IL-2) and Th2 profile (IL-4 and/or IL-5 and/or IL-13 and CD154) in participants who exhibited a positive CD4+ T-cell response at the respective timepoints. All participants with positive CD4+ T-cell responses were from the mRNA-1273 treatment groups.

**
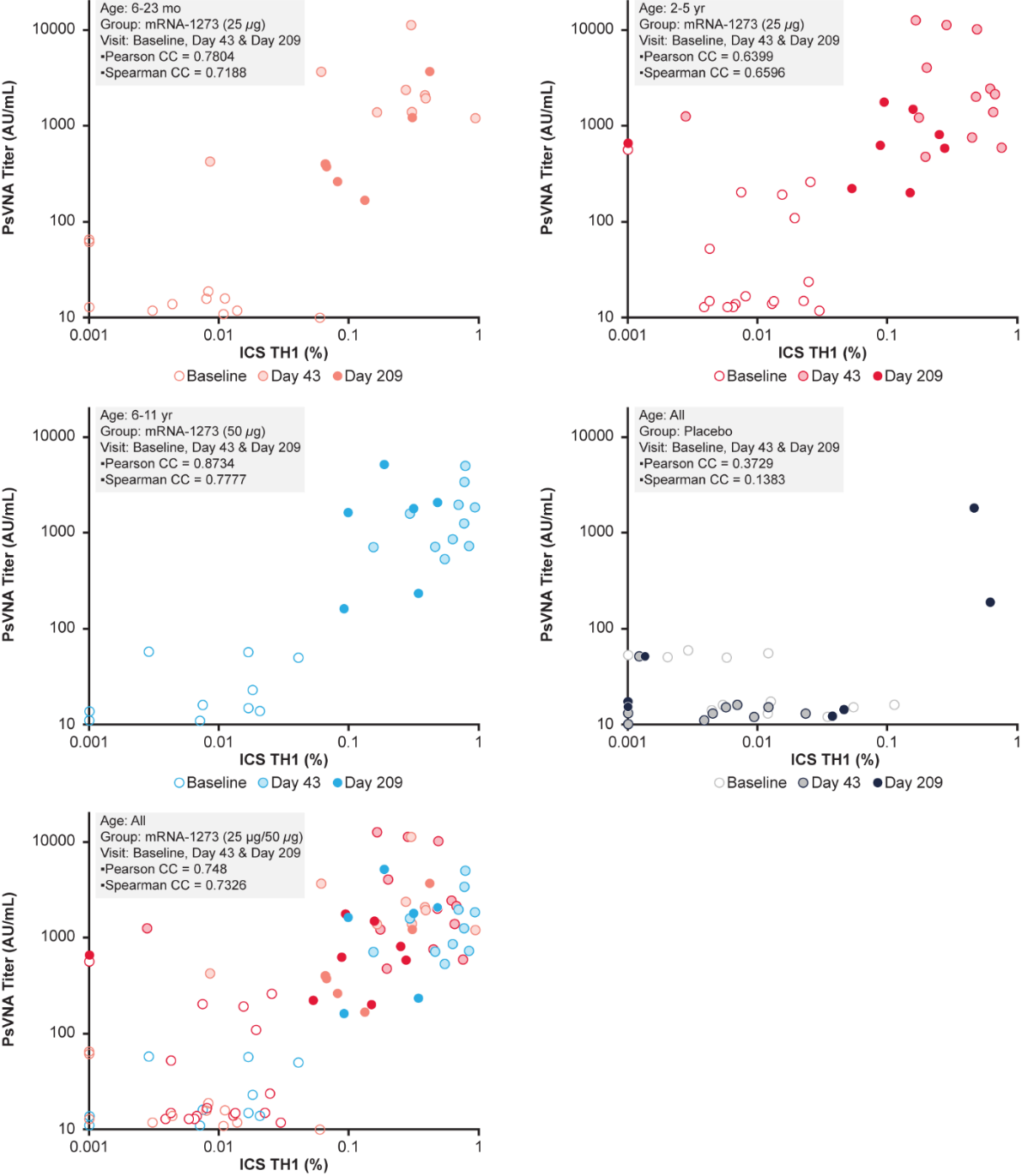
**

**Figure S3. Correlation between Th1 CD4+ responses and neutralizing antibodies across all age groups after vaccination with mRNA-1273.** nAbs against SARS-CoV-2 in mRNA-1273 (orange, ages 6-23 months; red, aged 2-5 years; blue, ages 6-11 years) or placebo (gray and black) groups as measured by PsVNA against SARS-CoV-2 S protein (strain D614G) plotted against frequencies of Th1 CD4+ T cells (expressing IFN-γ and/or IL-2) in mRNA-1273 or placebo groups, as measured by ICS assay. Correlation between these two assays was assessed by PCC of log-transformed results and SCC.
*Abbreviations*: AU, arbitrary units, ICS, intracellular cytokine stimulation; mo, month; PCC, Pearson Correlation Coefficient, PsVNA, pseudovirus neutralization assay; SCC, Spearman Rank Correlation Coefficient; Th, T helper cell; yr, year.

**(A)**

**(B)**

**Figure S4. Example flow cytometric gating strategy.
(A)** An example for the negative control (DMSO); **(B)** A response to the positive control (SEB). Within each set of plots, the upper 2 rows show the series of gates to identify CD4+ and CD8+ T cells, followed by 2 rows for the CD4+ T cell markers and the lower 2 rows for the CD8+ T cell markers. For the functional markers, one dimensional gates (referred to as marginal gates) are used followed by Boolean combinations for some markers (such as IFN-γ and/or IL-2 is the Boolean OR combination of IFN-γ and IL-2 gates). The number within each graph indicates the percentage of parent (such as each cytokine as a percentage of CD4+ or CD8+ T cells).

*Abbreviations*: DMSO, dimethyl sulfoxide; PBMC, peripheral blood mononuclear cell; SEB, staphylococcal enterotoxin B.
